# Synthesis and biological activity of novel 3-heteroaryl-2*H*-pyrido[4,3-*e*][1,2,4]thiadiazine and 3-heteroaryl-2*H*-benzo[*e*][1,2,4]thiadiazine 1,1-dioxides

**DOI:** 10.1007/s00706-013-0988-5

**Published:** 2013-05-14

**Authors:** Katarzyna Gobis, Henryk Foks, Jarosłw Sławiński, Ewa Augustynowicz-Kopeć, Agnieszka Napiórkowska

**Affiliations:** 1 Department of Organic Chemistry, Medical University of Gdańsk, Gdańsk, Poland; 2 Department of Microbiology, Institute of Tuberculosis and Pulmonary Diseases, Warsaw, Poland

**Keywords:** Sulfonamidine, Heterocycles, Synthesis, Anticancer activity, Structure–activity relationship

## Abstract

**Abstract:**

A series of novel 1,2,4-thiadiazine 1,1-dioxides were synthesized by condensation of 2-chlorobenzenesulfonamide and 4-chloropyridine-3-sulfonamide with heterocyclic methyl carbimidates obtained from heterocyclic carbonitriles and used at the time of their creation. Substituted amidines were isolated as the intermediates in the reaction with 2-chlorobenzenesulfonamide. Those intermediates were successfully cyclized to corresponding 1,2,4-thiadiazine 1,1-dioxides in pyridine with the addition of DBU. The newly synthesized compounds were evaluated for their tuberculostatic and anticancer activities. Eight compounds were able to inhibit the growth of some renal and non-small cell lung cancer cell lines.

**Graphical Abstract:**

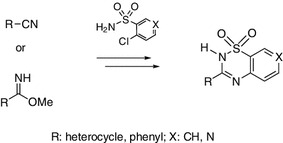

## Introduction

1,2,4-Benzothiadiazine 1,1-dioxides are well known for their cardiovascular and hypertensive effects [1, 2]. They also act as ATP-sensitive potassium channel openers, like their pyridyl analogs the 1,2,4-pyridothiadiazine 1,1-dioxides [3, 4]. Insulin release is inhibited as a result of that activity. Compounds of this group are the inhibitors of some enzymes, such as xanthine oxidase, HCV NS5B polymerase, and aldose reductase [5–7]. Benzothiadiazine 1,1-dioxides also constitute an important class of cyclic sulfonamides with broad-spectrum activity against bacteria, fungi, and *Mycobacterium tuberculosis* [8–10]. In an effort to develop new and effective chemotherapeutic agents for the treatment of tuberculosis, several series of heterocyclic compounds based on a sulfonamidine scaffold have recently been synthesized by our laboratory [11–13]. Here, we disclose the synthesis of novel carbimidate-derived 1,2,4-benzothiadiazine 1,1-dioxides and 1,2,4-pyridothiadiazine 1,1-dioxides with different heterocyclic rings at the 3-position. The synthesized compounds were screened for their antitubercular and anticancer activities in vitro.

## Results and discussion

The aim of the study was to investigate the reactivity of heterocyclic methyl carbimidates towards sulfonamides that possess a chlorine atom as a substituent at the *ortho* position to the sulfonamide group. The use of such sulfonamides facilitates the cyclization of sulfonated amidines, formed in the first stage of the reaction, to 1,2,4-thiadiazine 1,1-dioxides. The literature describes methods for the synthesis of 1,2,4-thiadiazine 1,1-dioxides. The most common method is the reaction of 2-aminobenzenesulfonamides with carboxylic acids, their halides, or anhydrides [14, 15]. Synthesis via the reaction of 2-aminosulfonamides with aldehydes is another method that has been used [16]. Other authors have reported the reaction of 2-halobenzenesulfonyl chlorides with amidines and aminopyridines in the presence of potassium carbonate [17]. The synthetic method in which substituted amidines react with TosNSO (*N*-sulfinyl-*p*-toluenesulfonamide) in acetic acid and hydrogen peroxide has also been described [18].

The method presented in this paper involved the use of heterocyclic methyl carbimidates as they are synthesized from the corresponding carbonitriles (Scheme 1). The carbimidates were reacted with 2-chlorobenzenesulfonamide and 4-chloropyridine-3-sulfonamide in methanol. We have previously described the diazabicyclo products of this reaction when it is carried out with a catalytic amount of DBU (1,8-diazabicyclo[5.4.0]undec-7-ene). This gave a linear amidine [13]. When DBU was equimolar to the sulfonamide, the reaction with 2-chlorbenzenesulfonamide led to linear structures **1**–**5**. However, when 4-chloropyridine-3-sulfonamide was used, the corresponding 3-heteroaryl-substituted pyrido[4,3-*e*][1,2,4]thiadiazine 1,1-dioxides **11**–**17** were the reaction products. Reducing the electron density on the carbon atoms of the pyridine ring at positions α and γ increases their vulnerability to nucleophilic attack. A halogen at the γ position of the pyridine ring is readily exchanged for a nucleophilic NH group. γ-Halopyridines are even more reactive than α-isomers [19]. Therefore, products that were cyclized to 1,2,4-thiadiazine 1,1-dioxides were easily obtained.

**Figure:**
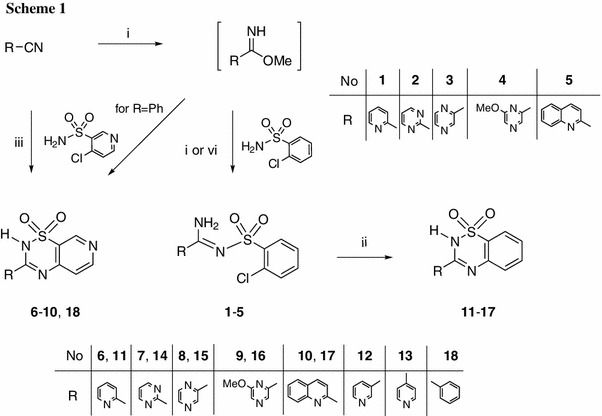
.

Using an equimolar amount of DBU in the case of methyl 6-chloropyrazinecarbimidate led to the creation of a mixture of linear and thiadiazine (**16**) structures that were quite easy to separate. However, the chlorine atom was replaced with a methoxy group.

Cyclization of amidines substituted with a 2-chlorobenzenesulfonamide moiety (**1**–**5**) to 3-heteroaryl-2*H*-benzo[*e*][1,2,4]thiadiazine 1,1-dioxides **6**–**10** was carried out by refluxing the substrates in pyridine in the presence of equimolar DBU. Cyclization did not occur in pyridine alone. The 3-phenylpyrido[1,2,4]thiadiazine derivative **18** was obtained from ethyl benzimidate hydrochloride and 4-chloropyridine-3-sulfonamide in methanol with excess DBU.

**Fig. 1:**
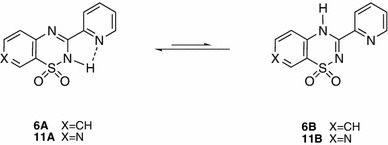
Structures of possible tautomers of compound **6**: 2*H* (**6A**) and 4*H* (**6B**)

The ^1^H NMR signals for the aromatic protons and NH-group protons were observed at 12–13 ppm. To elucidate the possible tautomeric forms of the representative compounds **6** (Fig. 1) and **11**, we estimated the total energies of the isolated molecules shown in Table 1.

**Table 1:** Calculated energies (*E*) and relative energies (Δ*E*) of tautomers **6A**–**6B** and **11A**–**11B**

Tautomer	Method	*E*/hartrees	Δ*E*/kJ mol^−1^
**6A** (2*H*)	RHF/6-31G*	−1170.176389	47.15
**6B** (4*H*)	RHF/6-31G*	−1170.158428	0
**6A** (2*H*)	B3LYP/6-31G*	−1175.525612	93.18
**6B** (4*H*)	B3LYP/6-31G*	−1175.490116	0
**11A** (2*H*)	RHF/6-31G*	−1186.167699	45.83
**11B** (4*H*)	RHF/6-31G*	−1186.150241	0
**11A** (2*H*)	B3LYP/6-31G*	−1191.559916	42.94
**11B** (4*H*)	B3LYP/6-31G*	−1191.543559	0

Energy values were calculated using ab initio RHF and B3LYP with the 6-31G* basis set

**Fig. 2:**
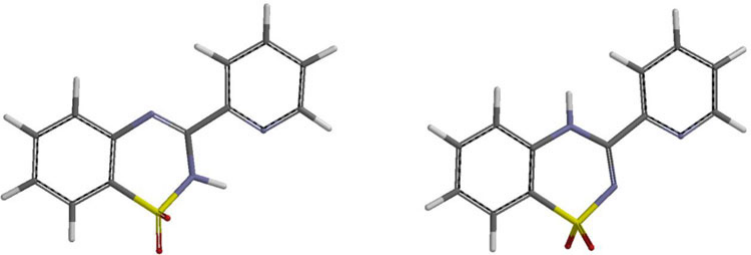
The optimized structures of the possible tautomers of compound **6** (calculated via the B3LYP/6-31G* method): 2*H* (*left*) and 4*H* (*right*)

Calculations were performed using ab initio Hartree–Fock and DFT methods in the gas phase. From the data presented in Table 1, one can infer that the 2*H* tautomers of compounds **6** and **11** are more energetically favorable than the 4*H* tautomers by 42.94–93.19 kJ/mol according to ab initio RHF as well as the density functional B3LYP method with the 6-31G* basis set [20]. Moreover, the possible optimized structures for compound **6** indicated conditions favoring hydrogen-bond formation between the hydrogen at nitrogen atom N-2 and the nitrogen atom of the pyridine substituent at carbon C-3. In this way, a stable five-membered cyclic structure can form, which additionally stabilizes that tautomer (Figs. 1, 2).

### Biological activity

Two of the 1,2,4-thiadiazine 1,1-dioxides obtained (**11**, **15**) were evaluated for their in vitro tuberculostatic activity against the *Mycobacterium tuberculosis* H_37_Rv strain and two “wild” strains isolated from tuberculosis patients: one (Spec. 210) resistant to *p*-aminosalicylic acid (PAS), isonicotinic acid hydrazide (INH), ethambutol (ETB), and rifampicin (RFP), and another (Spec. 192) that was fully sensitive to the tuberculostatics administered (Table 2). Isoniazid (INH) was used as a reference drug.

**Table 2:** Tuberculostatic activities of the newly synthesized compounds **11** and **15**

Compound	MIC/μg cm^−3^		
	H_37_Rv	Spec. 192	Spec. 210
**11**	50	50	25
**15**	100	50	25
INH	0.5	0.5	1.1

Minimum inhibitory concentrations for bacterial strains were determined by a twofold serial dilution method for microdilution plates and by a classical test-tube method of twofold successive dilution for mycobacterial strains. *M. tuberculosis* H_37_Rv, Spec. 192, Spec. 210

The tested compounds showed weak tuberculostatic activity, much lower than the reference INH (MIC 0.5–1.0 μg/cm^3^). The MIC values obtained when the compounds were tested against three strains ranged from 25 to 100 μg/cm^3^ for both compounds. Interestingly, both compounds were more active against the resistant 210 strain than the sensitive 192 one.

All of the newly synthesized compounds were also tested for antitumor activity. We previously described the synthesis of open sulfonamidine derivatives that are analogs of the 1,2,4-thiadiazine 1,1-dioxides presented here. We established that these compounds have notable antitumor activities [13]. It was interesting to see how the closure of the open structure to form the 1,2,4-thiadiazine 1,1-dioxide system affects this activity, especially considering that we have already reported the high affinity of the 1,2,4-thiadiazine 1,1-dioxide derivatives for isozyme CA IX (cancer-associated), an isoform of zinc enzyme carbonic anhydrase (CA, EC 4.2.1.1) [21], and the significant antitumor activities of these derivatives [22].

Compounds were tested in the framework of the Developmental Therapeutics Program (DTP) at the National Cancer Institute (Bethesda, MD, USA) on a panel of 60 human tumor cell lines derived from nine different cancer types: leukemia, lung, colon, CNS, melanoma, ovarian, renal, prostate, and breast. Among the compounds tested (**6**–**18**) in the preliminary NCI-60 one-dose screening test, eight of them (62 %) exhibited distinct growth inhibition (ΔGI) properties (Table 3). Six compounds (**6**, **9**, **10**, **12**, **13**, **16**) were active towards renal cancer cell lines: A498 (**6**, **10**), TK-10 (**9**, **16**), and UO-31 (**10**, **13**). These compounds inhibited the growth of those cell lines, with ΔGI ranging from 19.2 to 24.2 %. Derivative **10** exhibited activity against two renal cancer cell lines A498 and UO-31. Moreover, it was potent towards the colon cancer HT29 cell line (ΔGI 20.1 %). Derivatives **15** and **17** were active towards non-small cell lung cancer cell lines. Compound **15** was potent towards the HOP-92 cell line (ΔGI 25.9 %) and compound **17** was potent towards EKVX (ΔGI 19.2 %).

**Table 3:** One-dose screening data on the in vitro tumor growth inhibitory activities of compounds **6**–**18** at a dose of 10 μM

Compound	Mean growth values (MG_MID^a^)/%	Panel	Cell line	Growth inhibition ΔGI/%
**6**	103.8	Renal cancer	A498	19.2
**9**	102.1	Renal cancer	TK-10	19.7
**10**	102.9	Colon cancer	HT29	20.1
		Renal cancer	A498	21.7
			UO-31	51.1
**12**	104.3	Renal cancer	UO-31	21.7
**13**	102.3	Renal cancer	UO-31	24.5
**15**	101.8	Non-small cell lung cancer	HOP-92	22.0
**16**	101.4	Renal cancer	TK-10	24.2
**17**	95.1	Non-small cell lung cancer	EKVX	19.2

Data obtained from the NCI-60 DTP human tumor cell line screening

^a^ *MIG_MID* mean graph midpoint (i.e., the arithmetical mean growth for all tested cell lines)

## Conclusion

A series of novel 1,2,4-thiadiazine 1,1-dioxides with different six-membered nitrogen heterocyclic systems at the C-3 position were successfully synthesized by the reaction of heterocyclic methyl carbimidates with 2-chlorobenzenesulfonamide and 4-chloropyridine-3-sulfonamide. Substituted amidines were isolated as the intermediates in the reaction with 2-chlorobenzenesulfonamide. Those intermediates were successfully cyclized to the corresponding 1,2,4-thiadiazine 1,1-dioxides in pyridine with the addition of DBU. The syntheses of these new compounds were confirmed by analyzing their IR and NMR spectra as well as elemental analysis. The tuberculostatic and anticancer activities of the synthesized compounds were evaluated. The results showed that the synthesized 1,2,4-thiadiazine 1,1-dioxides exhibited rather poor tuberculostatic activities in vitro. Eight compounds (**6**, **9**, **12**, **13**, **15**–**17**) were able to inhibit the growth of some cancer cell lines derived mainly from renal cancer and non-small cell lung cancer.

## Experimental

All materials and solvents were of analytical reagent grade. Thin-layer chromatography was performed on Merck (Darmstadt, Germany) silica gel 60F_254_ plates and visualized with UV. Elemental analyses for C, H, N were performed on a Carlo Erba 1108 instrument (Thermo Scientific, Waltham, MA, USA) and the results for all of the obtained compounds were in agreement with calculated values to within ±0.3 %. NMR spectra in DMSO-*d*_6_ were recorded on Varian (Palo Alto, CA, USA) Unity Plus (500 MHz) and Gemini (200 MHz) instruments. IR spectra were determined as KBr pellets of the solids on a Satellite FT-IR spectrophotometer (Mattson Instruments, Madison, WI, USA). Melting points were determined with a Boethius apparatus (Franz Küstner Nachf. K.G., Dresden, Germany). The synthesis of sulfonylcarboximidamides **1**–**5** was described previously [13].

### *General method for the synthesis of 3*-*heteroaryl*-*2H*-*benzo[e][1,2,4]thiadiazine 1*-*1*-*dioxides* 6–10

The respective sulfonamide derivative **1**–**5** (5 mmol) was refluxed with 1.8 cm^3^ DBU (12 mmol) in 3 cm^3^ of pyridine for 2 h. The mixture was cooled down and 30 g of ice were added. The clear solution was acidified with glacial acetic acid. The precipitate was filtered off and purified by crystallization from a suitable solvent with activated carbon.

#### *3*-*(Pyridin*-*2*-*yl)*-*2H*-*benzo[e][1,2,4]thiadiazine 1,1*-*dioxide* (**6**, C_12_H_9_N_3_O_2_S)

This compound was recrystallized from dioxane, affording 0.791 g (61 %) of **6**. M.p.: 295–297 °C; IR (KBr): $\bar{v}$ = 3,268 (ν N–H), 3,066 (ν C–H), 1,615 (ν C=N), 1,595, 1,567 (ν C=C), 1,526 (*δ* N–H), 1,301, 1,173 (ν SO_2_), 826, 761 (γ C–H), 679, 555 (γ N–H), 499 cm^−1^; ^1^H NMR (200 MHz, DMSO-*d*_*6*_): *δ* = 7.52 (t, 1H, *J* = 7.3 Hz, Ph), 7.72–7.80 (m, 2H, 1H Ph and 1H pyridine), 7.86–8.00 (m, 2H, 1H Ph, 1H pyridine), 8.10 (t, 1H, *J* = 7.7 Hz, Ph), 8.32 (d, 1H, *J* = 7.3 Hz, pyridine), 8.85 (d, 1H, *J* = 4.4 Hz, pyridine), 12.62 (br s, 1H, NH + D_2_O exchangeable) ppm; ^13^C NMR (50 MHz, DMSO-*d*_*6*_): *δ* = 119.70, 122.01, 123.34, 123.54, 127.02, 127.93, 133.35, 135.79, 138.71, 148.24, 149.40, 152.23 ppm.

#### *3*-*(Pyrimidin*-*2*-*yl)*-*2H*-*benzo[e][1,2,4]thiadiazine 1,1*-*dioxide* (**7**, C_11_H_8_N_4_O_2_S)

This compound was recrystallized from a DMSO-dioxane mixture (1:1), affording 0.703 g (54 %) of **7**. M.p.: 307–310 °C; IR (KBr): $\bar{v}$ = 3,277 (ν N–H), 1,616 (ν C=N), 1,597, 1,568 (ν C=C), 1,525 (*δ* N–H), 1,410 (ν C=C), 1,302, 1,159 (ν SO_2_), 818, 766 (γ C–H), 675, 555 (γ N–H), 500 cm^−1^; ^1^H NMR (500 MHz, DMSO-*d*_*6*_): *δ* = 7.54 (t, 1H, *J* = 7.3 Hz, Ph), 7.76 (t, 1H, *J* = 7.3 Hz, Ph), 7.85 (t, 1H, *J* = 4.4 Hz, pyridine), 7.90–7.92 (m, 2H, Ph), 9.14 (d, 2H, *J* = 4.9 Hz, pyridine), 12.79 (br s, 1H, NH + D_2_O exchangeable) ppm; ^13^C NMR (50 MHz, DMSO-*d*_*6*_): *δ* = 119.32, 121.71, 123.69, 124.14, 127.42, 133.54, 135.33, 150.68, 156.74, 158.55 ppm.

#### *3*-*(Pyrazin*-*2*-*yl)*-*2H*-*benzo[e][1,2,4]thiadiazine 1,1*-*dioxide* (**8**, C_11_H_8_N_4_O_2_S)

This compound was recrystallized from a dioxane–ethanol mixture (1:1), affording 0.755 g (58 %) of **8**. M.p.: 275–278 °C; IR (KBr): $\bar{v}$ = 3,255 (ν N–H), 1,598, 1,570 (ν C=C), 1,526 (*δ* N–H), 1,481 (ν C=C), 1,304, 1,165 (ν SO_2_), 1,017 (*δ* C–H), 824, 773 (γ C–H), 596, 556 (γ N–H), 499 cm^−1^; ^1^H NMR (200 MHz, DMSO-*d*_*6*_): *δ* = 7.49–7.57 (m, 1H, Ph), 7.70–7.80 (m, 1H, Ph), 7.87–7.93 (m, 2H, Ph), 8.90-9.01 (m, 2H, pyrazine), 9.43-9.44 (m, 1H, pyrazine), 12.67 (br s, 1H, NH + D_2_O exchangeable) ppm; ^13^C NMR (50 MHz, DMSO-*d*_*6*_): *δ* = 119.57, 122.06, 123.63, 127.35, 133.53, 135.60, 143.98, 144.43, 148.52, 151.10 ppm.

#### *3*-*(6*-*Methoxypyrazin*-*2*-*yl)*-*2H*-*benzo[e][1,2,4]thiadiazine 1,1*-*dioxide* (**9**, C_12_H_10_N_4_O_3_S)

This compound was recrystallized from a dioxane–ethanol mixture (1:1), affording 0.755 g (52 %) of **9**. M.p.: 292–295 °C; IR (KBr): $\bar{v}$ = 3,298 (ν N–H), 1,601, 1,576, 1,548 (ν C=C), 1,522 (*δ* N–H), 1,392 (ν C=C), 1,303, 1,170 (ν SO_2_), 1,010 (*δ* C–H), 831, 765 (γ C–H), 672 (γ N–H), 502 cm^−1^; ^1^H NMR (500 MHz, DMSO-*d*_*6*_): *δ* = 7.54–7.58 (m, 1H, Ph), 7.75–7.95 (m, 3H, Ph), 8.66 (s, 1H, pyrazine), 8.96 (s, 1H, pyrazine), 12.20 (br s, 1H, NH + D_2_O exchangeable) ppm; ^13^C NMR (50 MHz, DMSO-*d*_*6*_): *δ* = 54.90, 119.61, 122.12, 123.67, 127.76, 133.48, 135.49, 136.01, 139.48, 140.53, 151.30, 159.25 ppm.

#### *3*-*(Quinolin*-*2*-*yl)*-*2H*-*benzo[e][1,2,4]thiadiazine 1,1*-*dioxide* (**10**, C_16_H_11_N_3_O_2_S)

This compound was recrystallized from dioxane–ethanol mixture (1:1), affording 0.619 g (40 %) of **10**. M.p.: 323–324 °C; IR (KBr): $\bar{v}$ = 3,441, 3,357, 3,242 (ν N–H), 2,957, 2,849 (ν C–H), 1,644, 1,596, 1,527 (ν C=C), 1,276, 1,136 (ν SO_2_), 1,084 (*δ* C–H), 828 (γ C–H), 556 (γ N–H) cm^−1^; ^1^H NMR (500 MHz, DMSO-*d*_*6*_): *δ* = 7.56 (t, 1H, *J* = 7.8 Hz, Ph), 7.78–7.83 (m, 2H, Ph), 7.93–7.99 (m, 2H, quinoline), 8.03 (d, 1H, *J* = 8.3 Hz, quinoline), 8.18 (d, 1H, *J* = 7.8 Hz, pyridine), 8.33–8.39 (m, 2H, quinoline), 8.70 (d, 1H, *J* = 8.3 Hz, quinoline), 12.54 (br s, 1H, NH + D_2_O exchangeable) ppm; ^13^C NMR (50 MHz, DMSO-*d*_*6*_): *δ* = 119.27, 119.51, 121.86, 123.69, 127.22, 128.58, 129.22, 129.43, 129.55, 131.33, 133.56, 135.30, 138.85, 146.50, 148.19, 152.00 ppm

### *General method for the synthesis of 3*-*heteroaryl*-*2H*-*pyrido[4,3*-*e][1,2,4]thiadiazine 1,1*-*dioxides* 11–18

The respective heteroarylcarbonitrile (5 mmol) was refluxed with 0.6 cm^3^ DBU (4 mmol) in 10 cm^3^ of methanol for 0.5 h. Then 0.77 g 4-chloropyridine-3-sulfonamide (4 mmol) were added and the mixture was refluxed for another 3 h. Methanol was removed under vacuum and 30 cm^3^ of water were added to the residue. The clear solution was acidified with glacial acetic acid. The precipitate was filtered off and recrystallized from a suitable solvent.

#### *3*-*(Pyridin*-*2*-*yl)*-*2H*-*pyrido[4,3*-*e][1,2,4]thiadiazine 1,1*-*dioxide* (**11**, C_11_H_10_N_4_O_2_S)

This compound was recrystallized from a dioxane–water mixture, affording 0.708 g (68 %) of **11**. M.p.: 331–333 °C; IR (KBr): $\bar{v}$ = 3,227 (ν N–H), 2,933 (ν C–H), 1,615 (ν C=N), 1,584, 1,497 (ν C=C), 1,305, 1,166 (ν SO_2_), 820, 742 (ν C–H, γ C–H), 605, 550 (γ N–H), 507 cm^−1^; ^1^H NMR (200 MHz, DMSO-*d*_*6*_): *δ* = 7.77–7.89 (m, 2H, pyridine), 8.11–8.19 (m, 1H, pyridine), 8.31–8.35 (m, 1H, pyridine), 8.77 (d, 1H, *J* = 5.9 Hz, pyridine), 8.86–8.88 (m, 1H, pyridine), 9.06 (s, 1H, pyridine), 12.93 (br s, 1H, NH + D_2_O exchangeable) ppm; ^13^C NMR (50 MHz, DMSO-*d*_*6*_): *δ* = 113.04, 118.10, 123.67, 128.51, 138.92, 141.75, 146.06, 147.32, 149.50, 152.93 ppm.

#### *3*-*(Pyridin*-*3*-*yl)*-*2H*-*pyrido[4,3*-*e][1,2,4]thiadiazine 1,1*-*dioxide* (**12**, C_11_H_8_N_4_O_2_S)

This compound was recrystallized from ethanol, affording 0.260 g (25 %) of **12**. M.p.: 300–303 °C; IR (KBr): $\bar{v}$ = 3,351 (ν N–H), 2,923, 2,808 (ν C–H), 1,617 (ν C=N), 1,579 (ν C=C), 1,508 (*δ* N–H), 1,482 ν C=C), 1,351, 1,299, 1,173 (ν SO_2_), 1,100 (*δ* C–H), 827, 808, 715 (γ C–H), 603, 551 (γ N–H), 511 cm^−1^; ^1^H NMR (200 MHz, DMSO-*d*_*6*_): *δ* = 7.50 (d, 1H, *J* = 5.7 Hz, pyridine), 7.64–7.70 (m, 1H, pyridine), 8.36–8.42 (m, 1H, pyridine), 8.76 (d, 1H, *J* = 5.8 Hz, pyridine), 8.86 (d, 1H, *J* = 4.8 Hz, pyridine), 9.07 (s, 1H, pyridine), 9.19 (s, 1H, pyridine), 12.50 (br s, 1H, NH + D_2_O exchangeable) ppm; ^13^C NMR (50 MHz, DMSO-*d*_*6*_): *δ* = 112.95, 118.08, 124.07, 128.18, 136.60, 142.81, 145.87, 149.36, 152.40, 153.64, 154.69 ppm.

#### *3*-*(Pyridin*-*4*-*yl)*-*2H*-*pyrido[4,3*-*e][1,2,4]thiadiazine 1,1*-*dioxide* (**13**, C_11_H_8_N_4_O_2_S)

This compound was recrystallized from DMSO, affording 0.437 g (42 %) of **13**. M.p.: >340 °C; IR (KBr): $\bar{v}$ = 3,057 (ν C–H), 1,629 (ν C=N), 1,489, 1,426 (ν C=C), 1,406, 1,287, 1,158 (ν SO_2_), 843, 688 (γ C–H), 601, 550 (γ N–H) cm^−1^; ^1^H NMR (500 MHz, DMSO-*d*_*6*_): *δ* = 7.54 (d, 1H, *J* = 5.9 Hz, pyridine), 7.99 (d, 2H, *J* = 4.9 Hz, pyridine), 8.77 (d, 1H, *J* = 5.4 Hz, pyridine), 8.89 (d, 2H, *J* = 5.4 Hz, pyridine), 9.10 (s, 1H, pyridine), 12.70 (br s, 1H, NH + D_2_O exchangeable) ppm; ^13^C NMR (50 MHz, DMSO-*d*_*6*_): *δ* = 113.34, 118.16, 122.26, 139.76, 143.25, 145.74, 150.72, 152.06, 154.78 ppm.

#### *3*-*(Pyrimidin*-*2*-*yl)*-*2H*-*pyrido[4,3*-*e][1,2,4]thiadiazine 1,1*-*dioxide* (**14**, C_10_H_7_N_5_O_2_S)

This compound was recrystallized from a DMSO–water mixture (1:1), affording 0.397 g (58 %) of **14**. M.p.: 326–329 °C; IR (KBr): $\bar{v}$ = 3,195, 3,160 (ν N–H), 1,623 (ν C=N), 1,589, 1,560, 1,503 (ν C=C), 1,297, 1,159 (ν SO_2_), 1,102 (*δ* C–H), 816 (γ C–H), 551 (γ N–H), 510 cm^−1^; ^1^H NMR (200 MHz, DMSO-*d*_*6*_): *δ* = 7.84 (d, 1H, *J* = 5.9 Hz, pyridine), 7.88 (t, 1H, *J* = 4.9 Hz, pyrimidine), 8.80 (d, 1H, *J* = 5.9 Hz, pyridine), 9.09 (s, 1H, pyridine), 9.16 (d, 2H, *J* = 4.9 Hz, pyrimidine), 13.06 (br s, 1H, NH + D_2_O exchangeable) ppm; ^13^C NMR (50 MHz, DMSO-*d*_*6*_): *δ* = 112.97, 118.17, 141.77, 143.25, 144.00, 144.66, 146.10, 148.93, 151.84, 153.02 ppm.

#### *3*-*(Pyrazin*-*2*-*yl)*-*2H*-*pyrido[4,3*-*e][1,2,4]thiadiazine 1,1*-*dioxide* (**15**, C_10_H_7_N_5_O_2_S)

This compound was recrystallized from ethanol affording 0.543 g (38 %) **15**. M.p.: 284–287 °C; IR (KBr): $\bar{v}$ = 3,204 (ν N–H), 2,923 (ν C–H), 1,631 (ν C=N), 1,588, 1,499 (ν C=C), 1,310, 1,161 (ν SO_2_), 1,021 (*δ* C–H), 812 (γ C–H), 604, 551 (γ N–H), 511 cm^−1^; ^1^H NMR (500 MHz, DMSO-*d*_*6*_): *δ* = 7.82 (d, 1H, *J* = 5.7 Hz, pyridine), 8.80 (d, 1H, *J* = 5.7 Hz, pyridine), 8.91-8.93 (m, 1H, pyridine), 9.02 (d, 1H, *J* = 2.4 Hz, pyrazine), 9.08 (s, 1H, pyrazine), 9.44 (s, 1H, pyrazine), 13.04 (br s, 1H, NH + D_2_O exchangeable) ppm; ^13^C NMR (50 MHz, DMSO-*d*_*6*_): *δ* = 112.93, 117.86, 124.43, 141.80, 146.20, 151.29, 152.95, 156.19, 158.62 ppm.

#### *3*-*(6*-*Methoxypyrazin*-*2*-*yl)*-*2H*-*pyrido[4,3*-*e][1,2,4]thiadiazine 1,1*-*dioxide* (**16**, C_11_H_9_N_5_O_3_S)

This compound was recrystallized from DMSO affording 0.886 g (52 %) **16**. M.p.: 285–286 °C (decomp.); IR (KBr): $\bar{v}$ = 3,283 (ν N–H), 1,615 (ν C = N), 1,590, 1,494 (ν C = C), 1,409, 1,392, 1,311, 1,169 (ν SO_2_), 1,005 (*δ* C–H), 894, 837, 710 (γ C–H), 603, 560 (γ N–H), 506 cm^−1^; ^1^H NMR (200 MHz, DMSO-*d*_*6*_): *δ* = 4.17 (s, 3H, OCH_3_), 7.78 (d, 1H, *J* = 5.8 Hz, pyridine), 8.67 (s, 1H, pyrazine), 8.80 (d, 1H, *J* = 5.8 Hz, pyridine), 8.97 (s, 1H, pyridine), 9.09 (s, 1H, pyrazine), 12.28 (br s, 1H, NH + D_2_O exchangeable) ppm; ^13^C NMR (50 MHz, DMSO-*d*_*6*_): *δ* = 55.43, 113.46, 118.68, 136.71, 140.25, 140.39, 146.53, 149.50, 152.41, 153.38, 159.70 ppm.

#### *3*-*(Quinolin*-*2*-*yl)*-*2H*-*pyrido[4,3*-*e][1,2,4]thiadiazine 1,1*-*dioxide* (**17**, C_15_H_10_N_4_O_2_S)

This compound was recrystallized from DMSO, affording 0.720 g (76 %) of **17**. M.p.: 347–349 °C; IR (KBr): $\bar{v}$ = 3,233 (ν N–H), 1,618 (ν C=N), 1,584, 1,489 (ν C=C), 1,321, 1,301, 1,161 (ν SO_2_), 770 (γ C–H), 591 (γ N–H), 515 cm^−1^; ^1^H NMR (200 MHz, DMSO-*d*_*6*_): *δ* = 7.78–7.82 (m, 1H, pyridine), 7.86–8.02 (m, 2H, quinoline), 8.17–8.20 (m, 1H, quinoline), 8.32–8.39 (m, 2H, 1H pyridine and 1H quinoline), 8.71 (d, 1H, *J* = 8.4 Hz, quinoline), 8.81 (d, 1H, *J* = 5.8 Hz, quinoline), 9.10 (s, 1H, pyridine), 12.81 (br s, 1H, NH + D_2_O exchangeable) ppm; ^13^C NMR (50 MHz, DMSO-*d*_*6*_): *δ* = 113.24, 118.08, 119.36, 128.63, 129.49, 129.59, 131.46, 136.48, 138.97, 141.91, 146.04, 146.47, 147.66, 152.76, 152.83 ppm.

#### *3*-*Phenyl*-*2H*-*pyrido[4,3*-*e][1,2,4]thiadiazine 1,1*-*dioxide* (**18**, C_12_H_9_N_3_O_2_S)

A mixture of 0.75 g ethyl benzimidate hydrochloride (4 mmol), 0.58 g 4-chloropyridine-3-sulfonamide (3 mmol), and 1.5 cm^3^ DBU (10 mmol) in 10 cm^3^ of dioxane was refluxed for 2.5 h. Then solvent was removed under vacuum and 30 cm^3^ of cold water were added to the residue. The mixture was acidified with 6 M HCl. The precipitate was filtered off and recrystallized from methanol, affording 0.506 g (65 %) of **18**. M.p.: 312–315 °C; IR (KBr): $\bar{v}$ = 3,274 (ν N–H), 3,089 (ν C–H), 1,609 (ν C=N), 1,490 (ν C=C), 1,323, 1,290, 1,158 (ν SO_2_), 1,096 (*δ* C–H), 821, 700 (γ C–H), 602, 543 (γ N–H), 511 cm^−1^; ^1^H NMR (200 MHz, DMSO-*d*_*6*_): *δ* = 7.53–7.77 (m, 4H, 3H Ph and 1H pyridine), 8.03–8.07 (m, 2H, Ph), 8.77 (d, 1H, *J* = 5.7 Hz, pyridine), 9.05 (s, 1H, pyridine), 8.73 (s, 1H, pyrazine), 12.45 (br s, 1H, NH + D_2_O exchangeable) ppm; ^13^C NMR (50 MHz, DMSO-*d*_*6*_): *δ* = 112.66, 117.99, 128.72, 129.21, 131.66, 133.55, 142.37, 145.98, 152.67, 155.84 ppm.

### Tuberculostatic activity

Investigations were performed by a classical test-tube method of successive dilution in Youmans’ modification of Proskauer and Beck’s liquid medium containing 10 % bovine serum [23, 24]. Bacterial suspensions were prepared from 14-day-old cultures of slow-growing strains and from 48-hour-old cultures of saprophytic strains [25, 26]. Solutions of the compounds in ethylene glycol were tested. Stock solutions contained 10 mg of the compounds in 1 cm^3^. Dilutions (geometric progression) were prepared in Youmans’ medium. A sample of the medium containing isoniazid (INH) as a reference drug but none of the investigated substances was used for comparison. Incubation was performed at a temperature of 37 °C. The MIC values were determined as the minimum concentration that inhibited the growth of the tested tuberculosis strains in relation to the probe with no tested compound.

### Anticancer activity

Compounds were tested at one concentration (10 μM). A mean graph midpoint (MG_MID) was calculated to give the average activity parameter over all cell lines. Cell lines that were insensitive in the screen were included in the calculate the MG_MID. Selectivity of a compound with respect to one or more cell lines of the screen was characterized by a high deviation of the particular cell line parameter from the MG-MID value. Details of the system and the information encoded by the activity pattern over all cell lines have been published [27–29].
